# Candidate Genes and MiRNAs Linked to the Inverse Relationship Between Cancer and Alzheimer’s Disease: Insights From Data Mining and Enrichment Analysis

**DOI:** 10.3389/fgene.2019.00846

**Published:** 2019-09-24

**Authors:** Cristina Battaglia, Marco Venturin, Aleksandra Sojic, Nithiya Jesuthasan, Alessandro Orro, Roberta Spinelli, Massimo Musicco, Gianluca De Bellis, Fulvio Adorni

**Affiliations:** ^1^Department of Medical Biotechnology and Translational Medicine (BIOMETRA), University of Milan, Segrate, Italy; ^2^Department of Biomedical Sciences, Institute of Biomedical Technologies–National Research Council (ITB-CNR), Segrate, Italy; ^3^Istituto Istruzione Superiore Statale IRIS Versari, Cesano Maderno, Italy

**Keywords:** genes, miRNAs, cancer, Alzheimer, inverse relationship, data mining, enrichment analysis, protein-protein interaction network

## Abstract

The incidence of cancer and Alzheimer’s disease (AD) increases exponentially with age. A growing body of epidemiological evidence and molecular investigations inspired the hypothesis of an inverse relationship between these two pathologies. It has been proposed that the two diseases might utilize the same proteins and pathways that are, however, modulated differently and sometimes in opposite directions. Investigation of the common processes underlying these diseases may enhance the understanding of their pathogenesis and may also guide novel therapeutic strategies. Starting from a text-mining approach, our *in silico* study integrated the dispersed biological evidence by combining data mining, gene set enrichment, and protein-protein interaction (PPI) analyses while searching for common biological hallmarks linked to AD and cancer. We retrieved 138 genes (ALZCAN gene set), computed a significant number of enriched gene ontology clusters, and identified four PPI modules. The investigation confirmed the relevance of autophagy, ubiquitin proteasome system, and cell death as common biological hallmarks shared by cancer and AD. Then, from a closer investigation of the PPI modules and of the miRNAs enrichment data, several genes (*SQSTM1*, *UCHL1*, *STUB1*, *BECN1*, *CDKN2A*, *TP53*, *EGFR*, *GSK3B*, and *HSPA9*) and miRNAs (miR-146a-5p, MiR-34a-5p, miR-21-5p, miR-9-5p, and miR-16-5p) emerged as promising candidates. The integrative approach uncovered novel miRNA-gene networks (e.g., miR-146 and miR-34 regulating p62 and Beclin1 in autophagy) that might give new insights into the complex regulatory mechanisms of gene expression in AD and cancer.

## Introduction

Cancer and dementia are complex pathologies that rise exponentially with age and dramatically affect quality of life and survival. Cancer and dementia are also described as the divergent manifestations of aging, the rival demons (Campisi, 2013), emerging from the opposite ends of a biological spectrum: uncontrolled cell proliferation characterizes carcinogenesis, whereas a progressive neuronal death marks neurodegeneration (López-Otín et al., 2013). Consistent epidemiological evidence suggests an intriguing inverse relationship between cancer and Alzheimer’s disease (AD) (Driver et al., 2012; Musicco et al., 2013; Romero et al., 2014; Freedman et al., 2016; Frain et al., 2017; Hanson et al., 2017). Four meta-analyses also support evidence on this hypothesis (Ma et al., 2014; Shi et al., 2015; Zhang et al., 2015; Catalá-López et al., 2017). Several theories and hypotheses have been proposed to address possible biological mechanisms underpinning this inverse association. For instance, in aging, cells undergo a metabolic reprogramming that leads to a divergent regulatory mechanism of metabolic pathways toward and away from respiration in mitochondria (Harris et al., 2014). In cancers, an elevation of aerobic glycolysis plays an important role in the biosynthesis of macromolecules and in promoting cell proliferation. In contrast, in senile neurodegeneration, in response to age-dependent diminished energy production, the decreased aerobic glycolysis is compensated by an increase in mitochondrial oxidative phosphorylation. The metabolic dysregulation along with the selective pressure to meet the cellular metabolic needs might be a common mechanism linking the two diseases and has suggested the attractive idea of repositioning of cancer drugs for AD treatment (Monacelli et al., 2016; Vargas et al., 2018). Among others, the theory of antagonistic pleiotropy (Campisi, 2013) offers a plausible interpretation of the two divergent manifestations of aging. This theory states that genes control more than one phenotype during the life span, and the same genes while promoting early-life benefits may exert negative actions in later life (Chinta et al., 2015). In the case of cancer and dementia, this biological trade-off implies that the processes that, in younger ages, stimulate cellular repair may boost neoplastic growth or limit cell regeneration if dysregulated in older ages. Similarly, the same processes leading to an increased rate of cell death may drive senile neurodegeneration while protecting from abnormal cellular reproduction.

A number of studies explored genes potentially involved in cancer and AD. Recently, three systematic reviews (Li et al., 2014; Shafi, 2016; Snyder et al., 2017) proposed sets of genes, pathways, and mechanistic and genetic links as possible actors in this complex biological context. Moreover, the molecular scenarios linking the two diseases were investigated in a few gene expression studies (Ibáñez et al., 2014; Klus et al., 2015; Sánchez-Valle et al., 2017). These studies indicated that a metabolic dysregulation of processes, such as mitochondrial metabolism and protein degradation, might play a dual role favoring the onset of cancer or AD. Noteworthy, the age is a relevant factor influencing all of the metabolic processes occurring in diseases, such as cancer and AD (Harris et al., 2014). Intriguingly, well-known genes associated with both diseases (e.g., *TP53*, *APOE*) have been recently associated with longevity traits (Tacutu et al., 2018). Apart from the age factor, the type of cell or tissue and other fine regulators, such as noncoding RNAs, impact biological processes underlying complex diseases, such as cancer and AD. Indeed, the role of miRNAs in complex diseases is now widely recognized (Hébert and De Strooper, 2009; Peng and Croce, 2016; Quinlan et al., 2017; Chen et al., 2018). MiRNAs exert pleiotropic effects because each miRNA can potentially target several mRNAs simultaneously, thereby influencing the expression of several genes and affecting a multitude of cellular pathways. As a consequence, miRNAs can have both beneficial and deleterious functions (antagonistic pleiotropy as, e.g., miRNA-34) (Liu et al., 2012). Because genes associated with antagonistic pleiotropy are likely to be evolutionarily retained due to their earlier beneficial functions, miRNA pathways could provide mechanisms to suppress their potentially deleterious age-related activities (Ahmad, 2017) but can also trigger the neurodegenerative process if they become dysregulated during aging (Godlewski et al., 2019). Hence, miRNAs together with their target genes might play a key role in the inverse association between cancer and AD.

The path of understanding the inverse relationship involves the exploration of the research from the field of –omics, the survey of the vast amount of cancer and AD literature, the investigation of the disease-specific databases, and the integration of cross-domain knowledge. The complexity of the cross-domain research might explain the fact that despite available knowledge on cancer and AD biology, only a few genes have been experimentally investigated with respect to the inverse relationship (Lee Houck et al., 2018). Recently, computational approaches have been developed to facilitate the exploitation of the huge amount of diverse biological data aiming at the prioritization of genes in human diseases (Moreau and Tranchevent, 2012). Text-mining approaches are effective in extracting biological information hidden in a massive amount of published biomedical articles and thus gaining insights on potential molecular biomarkers for complex diseases (Doncheva et al., 2012). Indeed, by means of literature mining and network analysis approaches, Bhasuran et al. (2018) have identified the functional association of genes in high-altitude diseases. Moreover, an integrative strategy that combined genomics data from various sources was instrumental to understand the molecular pathways involved in coronary artery diseases (Zhao et al., 2016). PubMed provides knowledge (information accompanied with evidence) that is generated and published across distributed sources, a massive amount of data that can be used to derive gene-disease associations. Thus, text-mining tools (e.g., Beegle; ElShal et al., 2016) have been proposed to simplify the process of extracting from PubMed and other sources (e.g., *Online Mendelian Inheritance in Man*: OMIM) the most relevant gene sets known to be linked to a given disease. The usage of computational methods empowered also functional analysis by integrating different databases (e.g., Gene Ontology, KEGG), which might help the process of discovery of new biological hypothesis. Interestingly, the availability of comprehensive atlas of the human tissue transcriptome enables the integration of data mining results with additional information about the expression levels of protein-coding genes across many tissues (*Genotype-Tissue Expression*, GTEx project) (Lonsdale et al., 2013). However, despite the effort of bioinformatics tools to integrate genomics data, the annotation of human genes or miRNAs for a specific purpose might be incomplete. To cope with this limitation, a manual annotation of candidate genes and miRNAs by cross-checking them in curated databases for both AD and cancer might be used. For AD, to our knowledge, two main databases are available: the *Integrative Database for Gene Dysregulation in Alzheimer’s Disease* (AlzBase) (Bai et al., 2016) and the catalogue of 430 genes reported to be associated with AD from 823 publications (Alzgset) (Hu et al., 2017). Focusing on cancer, two cancer databases can be used: the *Cancer Gene Census* (CGC) database (Forbes et al., 2010) and the Pathology Atlas included in the *Human Protein Atlas* (HPA) project (Uhlen et al., 2015). The *Transcriptome Wide Association Study* (TWAS) has been recently proposed to integrate gene expression measurements with genome-wide association studies (GWAS) to identify genes associated with complex traits, including AD and cancer (Gusev et al., 2016). Likewise, the *LongevityMap* database, based on GWAS, is available to pick genes associated with longevity trait in human populations (Tacutu et al., 2018).

The research strategy conducted in this study aimed at identifying candidate genes, miRNAs, and biological hallmarks shared by cancer and AD. To maximize the benefits of multiple studies and –omics approaches, which on their own provided dispersed pieces of knowledge on cancer and AD, we adopted a multistep methodological approach by combining automatic exploration of biomedical literature, annotation by means of evidence-based databases and gene set functional enrichment analysis.

## Methods

**Figure 1:**
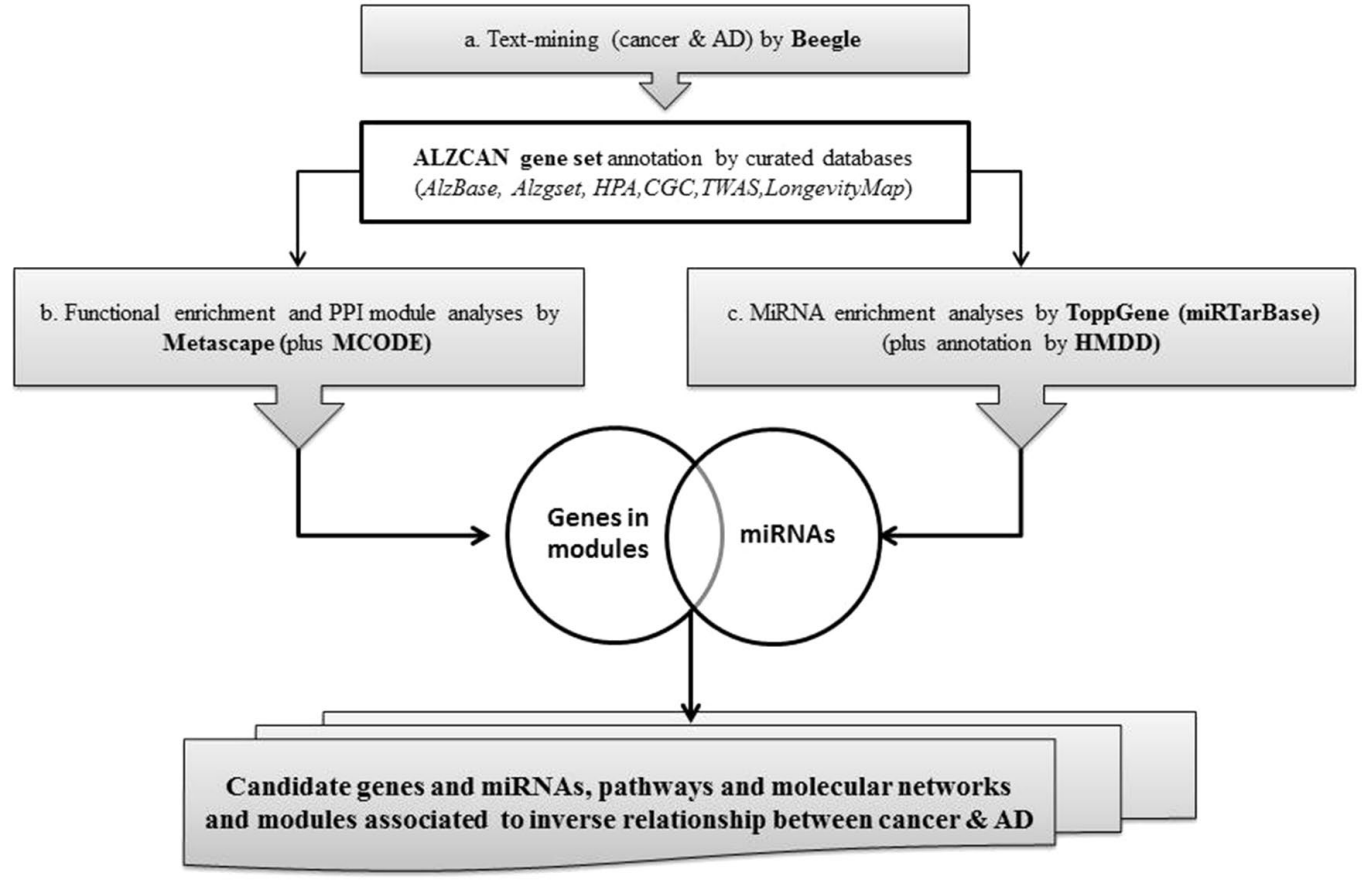
Flowchart of the bioinformatics strategy.

A concise scheme of the computational strategy is depicted in **Figure 1**. The computational strategy included three main steps: a) identification of a gene set by the text-mining tool Beegle and gene annotation by referring to specific databases and genomic resources; b) gene set functional enrichment analysis, followed by network analysis and protein-protein interaction (PPI) module reconstruction by means of Metascape; c) identification of gene-miRNA interactions specific for AD and cancer, particularly focusing on the analysis of PPI modules.

### Identification of Genes by Text-Mining and Annotation by Curated Databases

By means of a text-mining tool, Beegle, using the combined keywords “cancer OR cancers AND Alzheimer OR Alzheimer’s disease,” we retrieved a list of genes (namely, ALZCAN gene set) associated to cancer and AD. To complete the description of ALZCAN gene set, we cross-checked it with gene sets currently present in the AD and cancer-specific databases. For this aim, we queried two AD-specific databases, namely, AlzBase, referred to as a meta-analysis database, including gene expression studies comparing AD versus healthy subjects in different brain areas (http://alz.big.ac.cn/alzBase/ ) (Bai et al., 2016), and Alzgset, a catalogue of 430 genes reported to be associated with AD from 823 publications (Hu et al., 2017). To have a comprehensive annotation of gene expression levels, we referred to the GTEx database (GTEx v.6) (https://www.gtexportal.org/home/datasets ), which provides the expression level for 53 human tissues, including 12 different brain areas. Gene expression data as Reads Per Kilobase per Million mapped reads (RPKM) were downloaded from the UCSC Table Browser interface (https://genome.ucsc.edu/ ). By using Matlab R2018b “clustergram and heatmap” function (Matlab software, MathWorks^©^), the log10 transformed values (138 genes × 53 tissues) were visualized by a heat map applying an agglomerative hierarchical clustering method. For cancer information, we intersected the ALZCAN gene set with the CGC database (Forbes et al., 2010) (https://cancer.sanger.ac.uk/census ). We then downloaded the mRNA and protein expression data for both normal tissues and cancer types from HPA project v.18.1 (https://www.proteinatlas.org/ ). Cancer data, available in the Pathology Atlas, included a prognostic classification of human genes based on the correlation analyses of the mRNA expression levels and the clinical outcome of more than 17 different forms of human cancer. A gene was classified “prognostic” on the basis of the Kaplan-Meier survival analysis (p < 0.001). To assess the presence of associations between complex genetic traits (e.g., AD) and biological models (e.g., cancer types) for the genes being studied, TWAS resource (http://twas-hub.org/ ) was queried. We retained as statistically significant only the associations with Chi^2^ values ≤-3 or ≥3. To flag if the ALZCAN gene was associated to longevity, the LongevityMap database (build 3) (http://genomics.senescence.info/ ) was queried, limiting the selection to Caucasian populations.

### Functional Enrichment Analysis, Network, and PPI Module Reconstruction

To perform the pathway enrichment analysis and the gene network reconstruction, we used the Metascape tool (http://metascape.org ) (Tripathi et al., 2015) with the default parameters set. Imputing the gene set obtained from step a, pathway and enrichment analyses were carried out selecting the genomics sources: KEGG Pathway, GO Biological Processes, Reactome Gene Sets, Canonical Pathways, and CORUM (Giurgiu et al., 2019). All genes in the genome were used as the enrichment background. Terms with p < 0.01, minimum count 3, and enrichment factor >1.5 (the enrichment factor being the ratio between observed count and the count expected by chance) were collected and grouped into clusters based on their membership similarities. More specifically, p values were calculated based on accumulative hypergeometric distribution, q values were calculated using the Benjamini-Hochberg procedure to account for multiple testing. Kappa scores were used as the similarity metric when performing hierarchical clustering on the enriched terms, and then sub-trees with similarity >0.3 were considered a cluster. The most statistically significant term within a cluster was chosen as the one representing the cluster. To further capture the relationship among terms, a subset of enriched terms was selected and rendered as a network plot, where terms with similarity >0.3 are connected by edges. The terms with the best p values from each of the 20 clusters were selected, with the constraint that there were no more than 15 terms per cluster and no more than 250 terms in total. Subsequently, using Metascape default parameters, based on PPI enrichment analysis, we run a module network reconstruction based on the selected genomics databases. The resulting network was constructed containing the subset of proteins that form physical interactions with at least one other list member. Subsequently, by means of Molecular Complex Detection (MCODE) algorithm, we first identified connected network components, then a pathway and process enrichment analysis was applied to each MCODE component independently and the three best-scoring (by p value) terms were retained as the functional description of the resulting modules. The overall Metascape analysis allowed the identification of a list of significant overrepresented GO and KEGG categories, network plot, and PPI MCODE components (modules) linked to AD and cancer.

### Identification of Gene-miRNA Interactions Specific for AD and Cancer

To identify miRNAs that target the genes belonging to the ALZCAN gene set, first, we carried out a miRNA enrichment analysis by ToppGene (Chen et al., 2009) using default parameters. Then, from ToppGene results, we considered only the data related to the experimentally validated miRNA gene targets referred to the miRTarBase database (http://mirtarbase.mbc.nctu.edu.tw/ ) (Chou et al., 2018). A q value threshold of 0.05 after Bonferroni correction was applied for a given miRNA to be included in the enriched data set. Last, to further explore the involvement of a given miRNA in the pathogenesis of AD and cancer, we manually consulted the *Human microRNA Disease Database version 3.0* (HMDD v3.0) (http://www.cuilab.cn/hmdd ) (Li et al., 2014), which is a curated database that takes into account experiment-supported evidence for human microRNA and disease associations. By querying the database, we retrieved miRNAs associated with either cancer (any type of cancer) or AD, and we flagged them for belonging to both diseases. Then, by combining the results from steps a and b (**Figure 1**), we formulated novel gene-miRNA interactions.

## Results

### Description of ALZCAN Gene Set Linked to Both Cancer and AD

**Figure 2:**
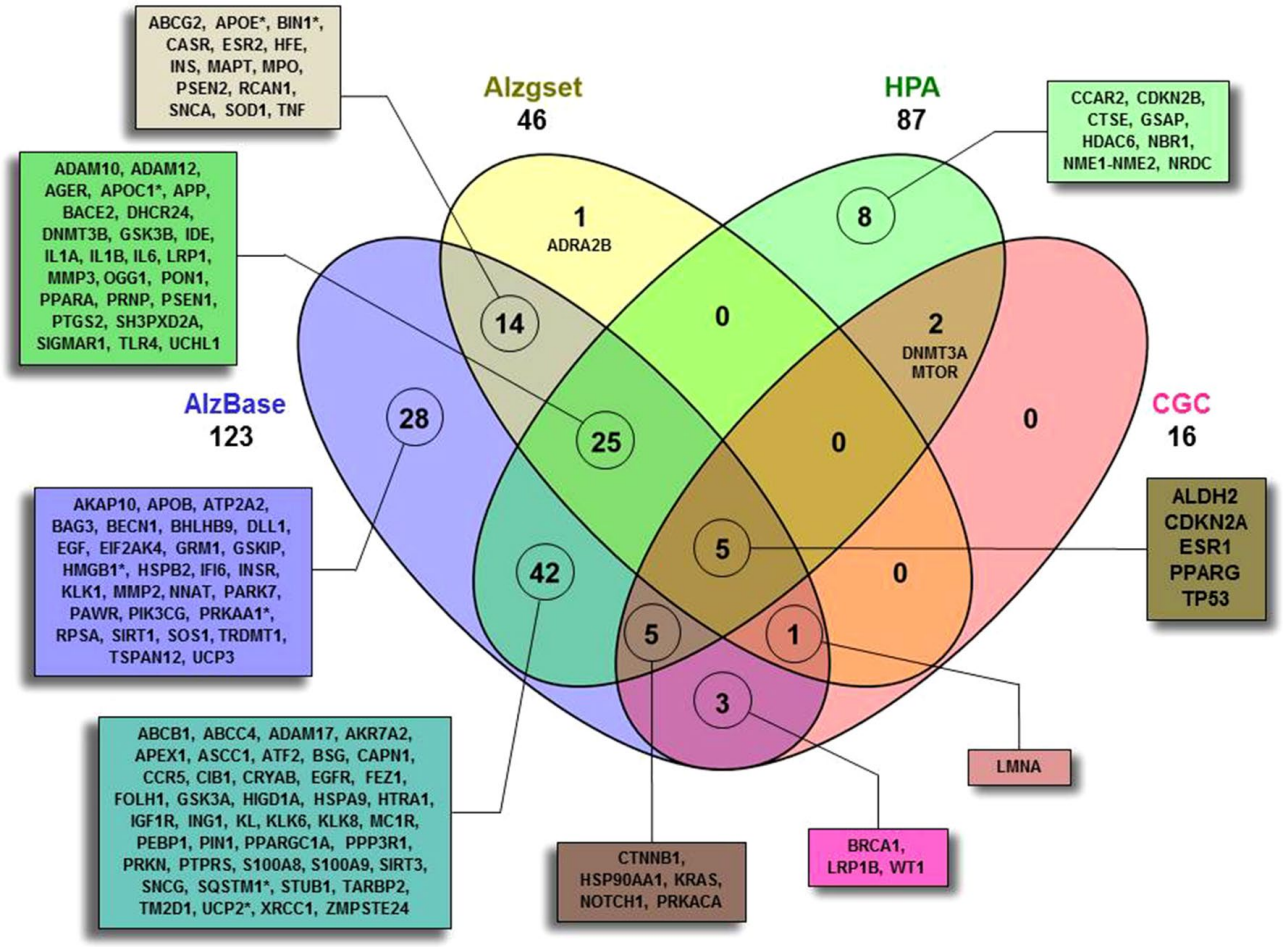
Four-set Venn diagram of the overlap between the ALCAN gene set with four databases. A total of 134 out of 138 were found to have an intersection with AlzBase (http://alz.big.ac.cn/alzBase/ ), Alzgset (Hu et al., 2017), prognostic genes on the basis of HPA (https://www.proteinatlas.org/ ), and CGC (https://cancer.sanger.ac.uk/census ). Four genes (*AKAP2*, *CAPN9*, *TKTL1*, and *WNT1*) were not found overlapping in any of the selected databases. (*)TWAS (http://twas-hub.org/ ) significantly trait-model associated genes. See **Supplementary Table S1** and **S3** for more details.

As a first step (**Figure 1**), by means of Beegle, we retrieved a total of 138 genes (ALZCAN gene set) that were further annotated by intersecting them with the AD databases (AlzBase and Alzgset) as well as cancer databases (CGC and HPA) (**Figure 2**, **Supplementary Table S1**). Only 4 out of 138 genes were not present in any of the selected databases. After querying of AlzBase, 123 out of 138 genes were computed as differentially expressed genes (DEG) with up and/or downmodulation (**Supplementary Table S1**). In particular, we found that the modulation of gene expression levels of *PIN1*, *UCHL1*, *PRKACA*, *CTNNB1*, *HSP90AA1*, *EGFR*, *NOTCH1*, *SQSTM1*, and *KRAS* was significantly altered in AD (**Supplementary Table S1**). Moreover, 46 out of 138 genes were also indicated by GWAS studies as associated with AD (Alzgset database). A total of 14 genes were included neither in AlzBase nor in Alzgset (**Figure 2**). By means of GTEx database, we retrieved the expression of the ALZCAN gene set across 53 human tissues (**Supplementary Table S2**). Referring to the 12 human brain tissues, on average, we found that out of 138, 39 genes displayed gene expression levels below 10 RPKM whereas 21 had gene expression levels over 50 RPKM (**Supplementary Table S2**). The clustering analysis by genes and tissues showed that the 12 human brain tissues clustered together (**Figure S1**). In the heat map, we recognized a few genes displaying an opposite tuning of expression in the cluster of brain tissues compared to others. For example, we observed high brain tissue expression levels for *FEZ1* and *UCHL1* genes compared to other tissues or, vice versa, very low brain expression levels for both *S100A9* and *S100A8* genes. We flagged 16 genes as Cancer Gene Census (**Figure 2**). By referring to the HPA data (**Supplementary Table S3**), 72 among out of 138 genes were classified as disease-related genes. According to the Pathology Atlas, 115 genes were detected at protein level in normal or cancer tissues and 87 out of 138 genes were associated with either favorable or unfavorable prognostic scores depending on cancer type (**Supplementary Table S3**). By means of TWAS, we recovered seven genes (*APOC1*, *APOE*, *BIN1*, *HMGB1*, *PRKAA1*, *SQSTM1*, and *UCP2*) significantly associated to AD traits and some cancer models (**Figure 2**, **Supplementary Table S1**). Notably, we found two genes displaying a negative association between *Alzheimer’s Disease (including proxy)* trait and *Lung Squamous Cell Carcinoma* model with Chi^2^ value of -18.3 (*APOE*) and -22.7 (*APOC1*) (see TWAS Trait association table at twas-hub.org/genes/APOE/and twas-hub.org/genes/APOC1/). Considering the LongevityMap database, we found 27 genes associated to longevity loci (**Supplementary Table S1**).

### Functional Enrichment Analysis Identified the Most Relevant Genes and Gene-Gene Modules

**Figure 3:**
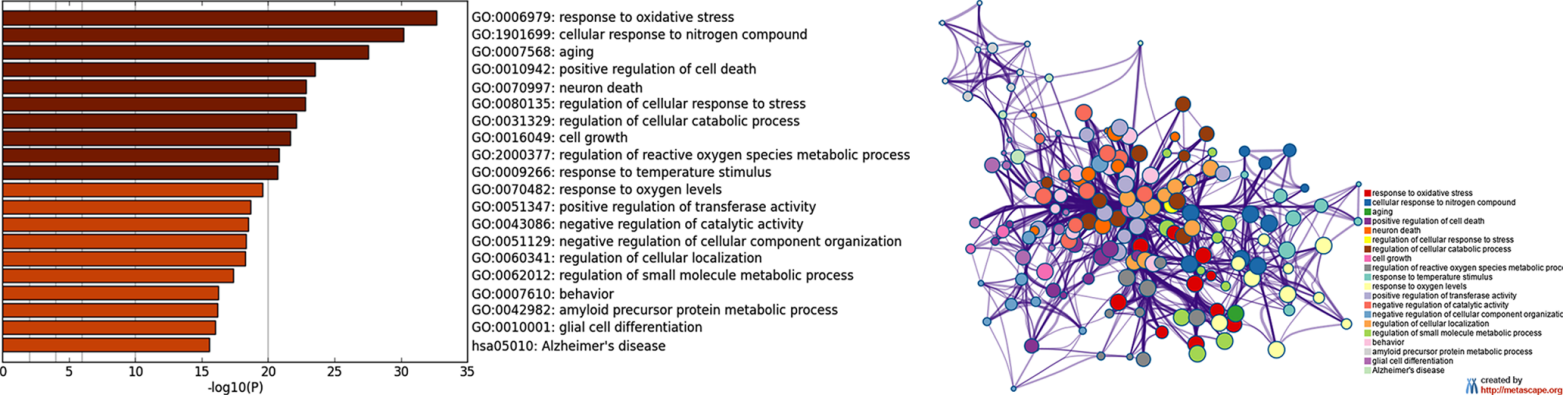
Functional enrichment analysis by Metascape. **(A)** Bar chart of clustered enrichment ontology categories (GO and KEGG terms); **(B)** enrichment ontology clusters including 124 genes. Each term is represented by a circle node, where its size is proportional to the number of input genes falling into that term, and its color represents its cluster identity (i.e., nodes of the same color belong to the same cluster). Terms with a similarity score >0.3 are linked by an edge (the thickness of the edge represents the similarity score). The network is visualized with Cytoscape (v3.1.2) with “force-directed” layout and with edge bundled for clarity. See **Supplementary Table S4** for more details.

As a second step (**Figure 1**), by means of Metascape, we found many overrepresented GO-BP terms (**Figure 3A**, **Supplementary Table S4**) and 124 out of 138 genes were included in a biological network (**Figure 3B**). Among the retrieved GO-BP terms, those related to *response to oxidative stress* (54 genes), *cellular response to nitrogen compound* (50 genes), *aging* (30 genes), *positive regulation of cell death* (45 genes) were the most significant. Within the enriched KEGG pathways, we found that the Alzheimer’s pathway (hsa05010) associated with 18 ALZCAN genes.

**Figure 4:**
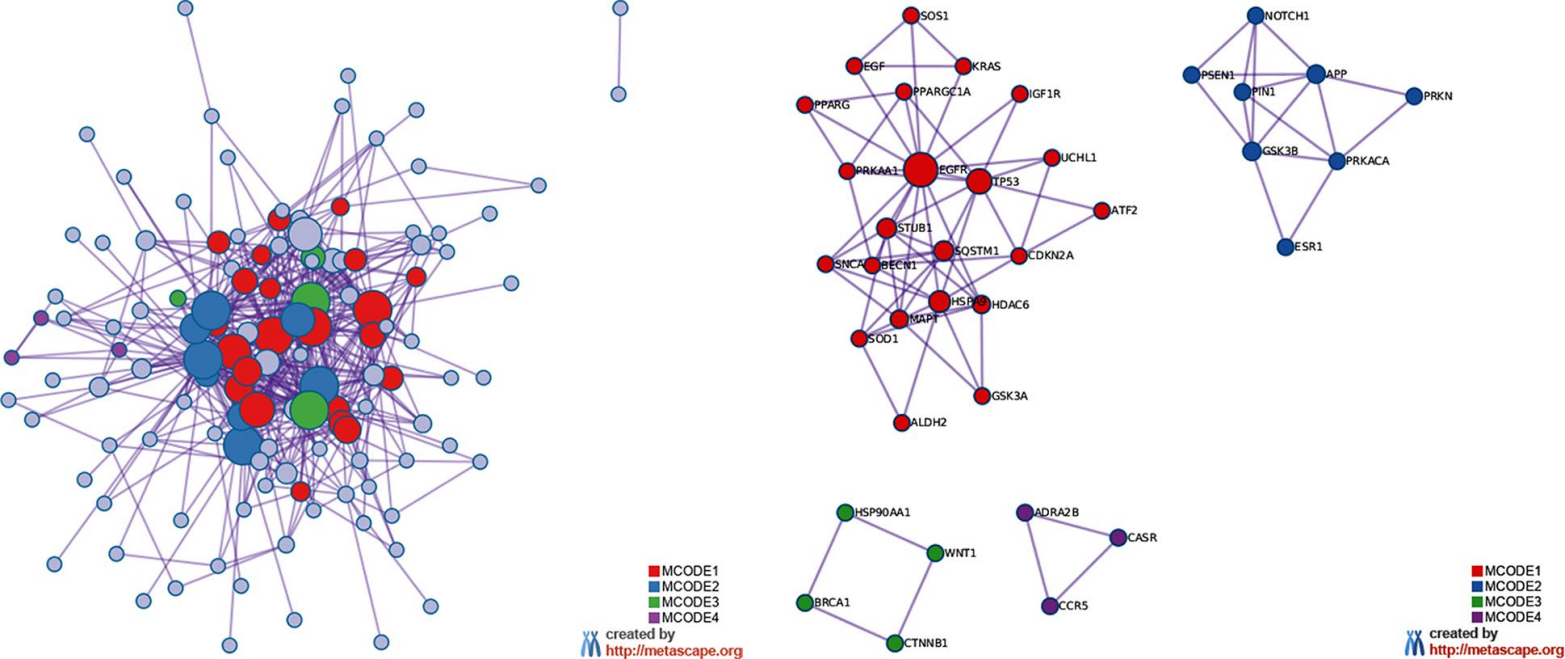
MCODE enrichment analysis by Metascape. **(A)** PPI interaction network. MCODE algorithm was applied to clustered enrichment ontology terms to identify neighborhoods where proteins are densely connected. Each MCODE network is assigned a unique color. **(B)** PPI MCODE component. GO enrichment analysis was applied to each MCODE network to assign “meanings” to the network component. See **Supplementary Table S5** for more details. Red, blue, green, and violet colors indicate modules 1, 2, 3, and 4, respectively.

The MCODE enrichment analysis based on PPI enrichment analysis resulted in a network (**Figure 4A**) characterized by the presence of four PPI modules, including 37 genes from which four genes, *EGFR*, *APP*, *WNT1*, *CCR5*, were defined as seed genes (**Figure 4B**, **Supplementary Table S5**). Among the top list of enriched terms of Module 1 (22 genes), we found three major GO-BP clusters, *positive regulation of programmed cell death, cellular response to oxidative stress, autophagy*, and two KEGG pathways, *glioma* and *longevity regulating pathway* (**Table 1**). The top list of enriched categories for Module 2 (8 genes) included *regulation of Wnt signaling pathway* (*APP*, *ESR1*, *GSK3B*, *NOTCH1*, *PRKN*, *PIN1*, and *PSEN1*) and *regulation of growth* (*APP*, *GSK3B*, *NOTCH1*, *PRKN*, and *PIN1*). Module 3 (4 genes) included *regulation of cellular response to stress* (*BRCA1*, *CTNNB1*, *HSP90AA1*, and *WNT1*) *and breast cancer* (*BRCA1*, *CTNNB1*, and *WNT1*). Module 4 (3 genes) was associated with *G alpha (i) signaling events* (*ADRA2B*, *CASR*, and *CCR5*).

**Table 1:** Top ranked clusters of enrichment terms of Module 1.

GO Biological Processes and KEGG terms*	Genes
GO:0043068-positive regulation of programmed cell death	*CDKN2A, ATF2, GSK3A, MAPT, PPARG, SNCA, SOD1, SOS1, TP53, SQSTM1, HDAC6, PPARGC1A*
GO:0034599-cellular response to oxidative stress	*EGFR, MAPT, PRKAA1, SNCA, SOD1, TP53, BECN1, HDAC6, PPARGC1A*
GO:0061919-process utilizing autophagic mechanism	*GSK3A, MAPT, PRKAA1, SNCA, TP53, UCHL1, BECN1, SQSTM1, HDAC6, PPARGC1A*
hsa05214: Glioma	*CDKN2A, EGF, EGFR, IGF1R, KRAS, SOS1, TP53*
hsa04211: Longevity regulating pathway	*ATF2, IGF1R, KRAS, PPARG, PRKAA1, TP53, PPARGC1A*

*Selected enrichment categories of Module 1. For more details, see **Supplementary Table S4**.

### Identification of Gene-MiRNA Interactions Linked to Both AD and Cancer

In the third step (**Figure 1**), the ALZCAN gene set was enriched with the information on specific miRNA targets. By means of ToppGene, we performed an enrichment analysis of the ALZCAN gene set for experimentally validated miRNA target sites included in the miRTarBase database. The analysis resulted in 90 significantly enriched miRNAs having functional miRNA-target interactions (MTIs) with the genes belonging to the gene set (**Supplementary Table S6**). For 61 of them, we found that strong experimental evidence (reporter assays, Western blot experiments, and qPCR assays) supported the MTIs. Moreover, according to the HMDD database, we found evidence referring to the involvement of 21 miRNAs to both AD and cancer (**Table 2**). Interestingly, among the most significantly enriched miRNAs, we retrieved those miRNAs or members of miRNA families that are most widely recognized as involved in AD pathogenesis, including miR-16-5p, miR-15a-5p, miR-15b-5p, miR-195-5p (all members belonging to the miR-15/107 family), miR-34a-5p, miR-181a-5p, miR-155-5p, miR-125b-5p, miR-9-5p, and miR-146a-5p (Femminella et al., 2015; Miya Shaik et al., 2018).

**Table 2:** List of 21 miRNAs linked to both AD and cancer according to HMDD^a^.

miRNA	ALZCAN (Gen)^b^	miRNA Targets
hsa-miR-16-5p	29 (1,557)	*KRAS, CDKN2A, ABCC4, HTRA1, CIB1, ASCC1, WT1, RPSA, TKTL1, APP, PTGS2, GSK3B, HIGD1A, ADRA2B, IGF1R, BRCA1, EGFR, BSG, MTOR, SQSTM1, AGER, PRKAA1, ALDH2, SNCG, TARBP2, HSPA9, PRNP, TP53, HSP90AA1*
hsa-miR-34a-5p	20 (735)	*CDKN2A, RCAN1, WNT1, CAPN1, BAG3, HMGB1, PPARA, XRCC1, DLL1, SIRT1, AKR7A2, IGF1R, PPP3R1, BRCA1, PIK3CG, TNF, CTNNB1, BECN1, NOTCH1, TP53*
hsa-miR-26b-5p	25 (1,874)	*CAPN9, TSPAN12, HTRA1, CIB1, STUB1, NRDC, FOLH1, PARK7, BAG3, INSR, HMGB1, LRP1B, PTGS2, GSK3B, IGF1R, PPP3R1, BRCA1, PIK3CG, PRKAA1, TLR4, PRKN, ADAM17, ABCG2, ATP2A2, TARBP2*
hsa-miR-15a-5p	16 (717)	*CDKN2B, ASCC1,WT1, TKTL1, APP, ATF2, GSK3B, HIGD1A, ADRA2B, BRCA1, BSG, UCP2, PRKAA1, SNCG, TARBP2, TP53*
hsa-miR-181a-5p	14 (553)	*KRAS, HDAC6, GSKIP, PTGS2, SIRT1, BRCA1, PEBP1, UCHL1, PRKN, ADAM17, ABCG2, CTNNB1, IL1A, NOTCH1*
hsa-miR-155-5p	16 (904)	*KRAS, CDKN2A, ABCC4, HTRA1, PSEN1, ADAM10, SIRT1, GSK3B, AKR7A2, EGFR, SH3PXD2A, PEBP1, DHCR24, AKAP10, CTNNB1, IL6*
hsa-miR-125b-5p	12 (431)	*CDKN2A, ABCC4, NRDC, S100A8, IGF1R, EGFR, AKAP2, TNF, MMP2, NME2, BACE2, TP53*
hsa-miR-9-5p	11 (350)	*ESR1, PPARA, SIRT1, GSK3B, LMNA, PRKAA1, LRP1, MMP2, BECN1, IL6, NOTCH1*
hsa-miR-195-5p	12 (639)	*INSR, ASCC1, TKTL1, APP, GSK3B, HIGD1A, ADRA2B, BSG, AGER, PRKAA1, SNCG, TARBP2*
hsa-miR-146a-5p	8 (102)	*PTGS2, BRCA1, EGFR, SQSTM1, TLR4, IL6, NOTCH1, SOS1*
hsa-miR-17-5p	15 (1,181)	*ESR2, APEX1, HMGB1, RPSA,APP, AKR7A2, PPP3R1, SQSTM1, TNF, MMP2, NBR1, HSPB2, PRNP, TP53, HSP90AA1*
hsa-miR-27a-3p	10 (429)	*KRAS, APEX1, PPARG, GSK3B, EGFR, PIK3CG, SNCG, NME2, TP53, SOS1*
hsa-miR-15b-5p	12 (760)	*INSR, ASCC1, TKTL1, APP, GSK3B, HIGD1A, ADRA2B, BSG, PRKAA1, SNCG, ATP2A2, TARBP2*
hsa-miR-21-5p	11 (611)	*STUB1, HMGB1, PPARA, ATF2, ABCB1, IGF1R, BRCA1, EGFR, TLR4, MMP2, IL1B*
hsa-miR-142-3p	9 (389)	*ESR1, HMGB1, XRCC1, PIK3CG, ABCG2, CTNNB1, IL1A, ATP2A2, IL6*
hsa-miR-26a-5p	9 (457)	*ESR1, PTGS2, GSK3B, BRCA1, PIK3CG, PRKAA1, ADAM17, IL6, DNMT3B*
hsa-miR-100-5p	7 (251)	*ESR1, APEX1, HMGB1, IGF1R, MTOR, BECN1, ATP2A2*
hsa-let-7b-5p	13 (1,215)	*NRDC, RCAN1, SIGMAR1, HMGB1, RPSA, PTGS2, GSK3A, PPARGC1A, IGF1R, SH3PXD2A, TLR4, ATP2A2, HSP90AA1*
hsa-miR-7-5p	9 (578)	*SIGMAR1, IDE, AKR7A2, IGF1R, EGFR, SQSTM1, PIK3CG, TLR4, SNCA*
hsa-miR-375	8 (477)	*CDKN2B, IGF1R, PEBP1, CTNNB1, IFI6, TP53, HSP90AA1, DNMT3B*
hsa-miR-181c-5p	6 (291)	*KRAS, GSKIP, SIRT1, PEBP1, ADAM17, IL1A*

^a^HMDD, Human MicroRNA Disease Database (HMDD v3.0); ^b^number of genes of ALZCAN gene set (total number of genes in the genome according to ToppGene). For more details, see **Supplementary Table S6**.

Limiting the enrichment analysis to the 37 genes belonging to the four modules, we highlighted subsets of 13, 9, and 10 significantly enriched miRNAs with functional MTIs for modules 1, 2, and 3, respectively (**Table 3**). No significant miRNAs were found for genes within Module 4. Notably, all miRNAs but miR-34a-5p, which has target genes in both modules 1 and 3, were module-specific. According to HMDD v3.0, among the 13 enriched miRNAs targeting genes belonging to Module 1, eight miRNAs (namely, miR-16-5p, miR-27a-3p, miR-206, miR-125b-5p, miR-7-5p, miR-146a-5p, miR-21-5p, and miR-34a-5p) have been associated to both AD and cancer. In addition, miR-9-5p and miR-296-5p in Module 2, as well as miR-139-5p and miR-375 in Module 3, have been linked to AD and cancer (**Table 3**). It is worth noting that all of the eight miRNAs in Module 1 that are involved in both AD and cancer, apart from miR-34a-5p, share *EGFR* as a validated target gene; *IGF1R* is targeted by six miRNAs, and *TP53* is targeted by four miRNAs. Last, a third receptor gene, *NOTCH1*, is the target of miR-9-5p and miR-296-5p, the two enriched miRNAs in Module 2 with involvement in AD and cancer.

**Table 3:** List of miRNAs and their target genes belonging to PPI modules.

PPI module	miRNA ID	Target genes (miRTarBase)
Module 1	**hsa-miR-16-5p**	*EGFR, CDKN2A, KRAS, SQSTM1, HSPA9, TP53, IGF1R, ALDH2, PRKAA1*
	hsa-miR-30a-5p	*STUB1, EGFR, BECN1, TP53, IGF1R, PRKAA1*
	**hsa-miR-27a-3p**	*EGFR, KRAS, TP53, PPARG, SOS1*
	**hsa-miR-206**	*KRAS, SOD1, IGF1R*
	**hsa-miR-125b-5p**	*EGFR, CDKN2A, TP53, IGF1R*
	hsa-miR-125b-1-3p	*TP53, IGF1R*
	**hsa-miR-7-5p**	*EGFR, SQSTM1, IGF1R, SNCA*
	**hsa-miR-146a-5p**	*EGFR, SQSTM1, SOS1*
	**hsa-miR-21-5p**	*STUB1, EGFR, ATF2, IGF1R*
	hsa-miR-548e-5p	*BECN1, SQSTM1, PRKAA1*
	hsa-miR-877-5p	*KRAS, SQSTM1, UCHL1*
	**hsa-miR-34a-5p**	*CDKN2A, BECN1, TP53, IGF1R*
	hsa-miR-150-3p	*TP53, IGF1R*
Module 2	**hsa-miR-9-5p**	*NOTCH1, ESR1, GSK3*B
	hsa-miR-744-5p	*PIN1, GSK3*B*, PRKACA*
	hsa-miR-1910-5p	*ESR1, GSK3*B
	hsa-miR-296-5p	*NOTCH1, PIN1*
	hsa-miR-874-3p	*ESR1, PIN1*
	hsa-miR-6073	*ESR1, APP*
	hsa-miR-935	*NOTCH1, PSEN1*
	hsa-miR-4709-3p	*ESR1, APP*
	hsa-miR-22-3p	*ESR1, PRKACA*
Module 3	**hsa-miR-34a-5p**	*BRCA1, CTNNB1, WNT1*
	**hsa-miR-139-5p**	*HSP90AA1, WNT1*
	hsa-miR-152-3p	*HSP90AA1, WNT1*
	hsa-miR-548l	*HSP90AA1, CTNNB1*
	hsa-miR-148a-3p	*HSP90AA1, WNT1*
	hsa-miR-1226-3p	*HSP90AA1, CTNNB1*
	hsa-miR-1826	*CTNNB1*
	hsa-miR-101-3p	*HSP90AA1, CTNNB1*
	hsa-miR-148b-3p	*HSP90AA1, WNT1*
	**hsa-miR-375**	*HSP90AA1, CTNNB1*

Bold, miRNA associated with both Alzheimer’s disease and cancer based on the Human microRNA Disease Database (HMDD v3.0).

## Discussion

The biological understanding of the inverse occurrence between the two diseases, widely supported by epidemiological studies, is still an open question. In recent years, a significant progress has been made in exploring the molecular mechanisms of both AD and cancers. Nowadays, by means of the advancements in integrative bioinformatics, we can exploit the massive amount of -omics data and biological information to tackle the complex nature of both these age-related diseases on much larger scales. In this study, by mining of biomedical literature and by delineating the interconnection of genes by means of pathway-based and network-based analyses, we retrieved a pool of 138 genes (ALZCAN gene set) and their interactions, thus obtaining valuable data for further analysis on their relationship with both cancer and AD. Moreover, by retrieving miRNAs targeting the ALZCAN gene set, we postulated some promising miRNA-gene interrelationships evidencing the complex gene expression regulation that might be shared between the two diseases. This integrated bioinformatics approach, by focusing on the biological function of genes, pathways, and network analysis, was not only instrumental in yielding a more comprehensive view of biological processes but also more robust in terms of the influence of false-positive genes that might be retrieved by means of basic automatic text-mining procedures.

As revealed by functional enrichment analysis, we observed that 124 out of 138 ALZCAN genes are densely interconnected in many metabolic pathways and cellular processes (**Figure 3B**). Terms such as *response to oxidative stress*, *cellular response to nitrogen compound*, *aging*, and *positive regulation of cell death* were overrepresented in the ALZCAN gene set. Interestingly, many of these processes have been recently enlisted by Lee Houck et al. (2018) that reviewed the biological and genetic overlap between cancer and neurodegeneration. As expected, because of the presence of an emerging body of scientific literature, we found that *TP53*, *PIN1*, and *APP* genes were top-ranked by text-mining *via* Beegle (**Supplementary Table S1**), confirming them as important players in the inverse association between cancer and AD (Shafi, 2016; Feng et al., 2017; Galvão et al., 2019). The scenarios underlying the onset of both AD and cancer are however pretty complicated, and many factors (e.g., aging, oxidative stress, inflammation) are affecting the cell performance in specific tissues. Of note, amid the biological processes highlighted by our study, we found processes implicated in the cellular homeostasis and adaptation to stress. The human tissues combat age-related diseases, such as cancer and neurodegeneration by enhancing intracellular processes linked to the protein turnover pathways (e.g., proteolysis and autophagy) by which cellular components are degraded and recycled (Barbosa et al., 2018). The protein degradation process, responsible for the correct protein clearance, is a cooperating system between the ubiquitin-proteasome system (UPS) and autophagy for maintaining protein homeostasis, particularly within postmitotic cells (nervous system cells) (Vilchez et al., 2014; Leidal et al., 2018). It is important to highlight that aging is a manifestation of the accumulation of cellular damage over time that is, in turn, depending on nine hallmarks, including loss of proteostasis, reactive oxygen species, genome instability (López-Otín et al., 2013). Hallmarks of aging are significant with respect to both cancer and AD. In the brain, impaired neuronal autophagy promotes the accumulation of toxic protein aggregates and damaged organelles linked to dementia. Intriguingly, autophagy has been shown to be important for multiple aspects of cancer biology, including cell metabolism, protein and organelle turnover, and cell survival (Santana-Codina et al., 2017). Both the enhancement and inhibition of autophagy have been suggested as therapeutic strategies in cancer. Noteworthy, a recent transcriptomics meta-analysis conducted comparing AD and three cancer types indicated protein degradation as a candidate biological process orchestrating the comorbidity between AD and cancer (Ibáñez et al., 2014). In addition, a meta-analysis conducted by taking into account the physicochemical properties of gene products involved in cancer and senile dementias strongly supported the role of protein unfolding as trade-off factors triggering pathological conditions (Klus et al., 2015).

By means of gene-gene network reconstruction analysis, we found four functional modules that rely on the crosstalk between several biological processes and pathways (**Figure 4B**). In Module 1, we found 10 genes that play a role in protein turnover system that includes autophagy and UPS (**Table 1**, **Supplementary Table S5**). Here, we focused the discussion on five genes: *SQSTM1*, *UCHL1*, *STUB1*, *BECN1*, and *HSPA9*. *SQSTM1/p62* (sequestosome 1) is an autophagy receptor required for selective macroautophagy and functions as a bridge between polyubiquitinated cargo and autophagosomes. *SQSTM1/p62* also serves as a signaling hub for multiple pathways associated with neurodegeneration, providing a potential therapeutic target in the treatment of neurodegenerative diseases (Ma et al., 2019). By database cross-checking, we found that *SQSTM1/p62* is classified as a disease-related gene and is upregulated in AD (according to AlzBase). Intriguingly, the upregulation and/or inefficient degradation of p62 has been linked with tumorigenesis (Zhang et al., 2019). By means of TWAS, we retrieved significant data on expression quantitative trait locus (eQTL) for *SQSTM1*, finding negative associations (Chi^2^ value equal to -3) between AD trait and two tissue models, *Brain Prefrontal Cortex* and *Thyroid Carcinoma*, thus suggesting a link between the gene expression variation and the genotypes, deserving further investigation with respect to the inverse relationship between cancer and AD. *SQSTM1*/*p62* is gaining attention because it is involved in dementia and cancers (Bitto et al., 2014; Taniguchi et al., 2016). *UCHL1* is an ubiquitin-protein hydrolase involved in the processing of both ubiquitin precursors and ubiquitinated proteins. Although it is highly expressed in human brain tissues (GTEx database, **Figure S1**), *UCHL1* is implicated in neurodegenerative diseases (Bishop et al., 2016). This gene is also found consistently downregulated in AD (AlzBase data) but, conversely, upregulated in cancers with a prognostic score (**Supplementary Table S3**). Recently, more attention has been paid to the relationship between malignancies and the UCH family, which plays different roles in the progression of several tumors (Fang and Shen, 2017). *STUB1(CHIP)* gene encodes for an E3 ubiquitin-protein ligase, ubiquitously expressed in human tissues, targets misfolded chaperone substrates toward proteasomal degradation and modulates the activity of several chaperone complexes, including Hsp70, Hsc70, and Hsp90. *STUB1* has also emerged as a hub gene in the regulation of biosynthetic processes in the brain of the accelerated senescence mice (SAMP8) used as a robust model of AD (Cheng et al., 2013). The role of *STUB1* in human malignant disorders has been reviewed by Cao et al. (2016). Although the modulation of *STUB1* expression in AD and cancers is controversial, it might be an interesting gene deserving to be further investigated. Concerning the molecular processes involved in controlling protein folding, we also found BECN1 (Beclin 1), a protein involved in the regulation of autophagy, which has been found reduced in patients with AD (Jaeger et al., 2010). Notably, the gene expression level of *BECN1* is found significantly altered in cancer and AD but in opposite manner (Ibáñez et al., 2014). Beclin1 might act as a protein platform interlinking autophagy and apoptosis (Kang et al., 2011) and, in turn, might be linked to inflammation (Salminen et al., 2013). The relevance of Beclin1, as well as autophagy, is a matter of ongoing debate in cancer therapy (Toton et al., 2014; Galluzzi et al., 2015). Together with *STUB1* and *UCHL1*, our analysis highlighted a member of the heat shock protein family, *HSPA9* (Mortalin), which plays a key role in preventing protein misfolding and aggregation. Its gene expression is oppositely regulated in AD and cancer. Lately, Mortalin has been suggested as a potential therapeutic target for AD (Ou et al., 2014). In the cancer field, findings support its role in the induction of epithelial-mesenchymal transition (EMT), prompting further investigation of its therapeutic value for contrasting metastasis (Na et al., 2016). Altogether, this supporting evidence suggests that protein clearance system in age-associated diseases might be attractive multifaceted molecular machinery, with therapeutic potential for both cancer and AD.

Among the enriched biological processes of Module 1, we also obtained the terms *aging* and *positive regulation of cell death*, including *CDKN2A*, *TP53*, *EGFR* genes that play a pivotal biological role. These genes are well-known molecular players in cell proliferation, survival, adhesion, and apoptosis (LaPak and Burd, 2014). By manual annotation, we found that *CDKN2A* and *TP53* were present in all databases (AlzBase, Alzgset, CGC, and HPA) (**Figure 2**). An increased level of *CDKN2A* (cyclin-dependent kinase inhibitor 2A) resulted in both brain and blood cells from APP/PS1 mice (Esteras et al., 2012). Interestingly, linkage and association studies linked the CDKN2A locus (9p21.3) to late-onset AD (LOAD study) families (Züchner et al., 2008). The gene is upregulated in AD (AlzBase) as well as in cancer. The tumor protein p53 (*TP53*) is one of the best-known hallmarks of cancer and has been linked to the longevity trait in the human population as well as to AD (Lanni et al., 2012). *TP53* network has been suggested as a candidate signaling cascade linked to the inverse relationship between cancer and AD. *EGFR* (epidermal growth factor receptor) is widely recognized for its importance in cancer and has been catalogued as an oncogene. Recently, a pivotal role of *EGFR* in AD has been proposed (Shafi, 2016). The finding of the overexpression of *EGFR* in AD (AlzBase) is however in contrast with its absence in the central core of AD neuritic plaques (Birecree et al., 1991). A polymorphism of this gene has been associated with glioma (Sanson et al., 2011). Whether this gene polymorphism could influence its transcriptional activity in the etiology of AD deserves further investigation.

Besides the multiple factors implicated in AD and cancer, increasing evidence points toward a role of Wnt signaling in the etiology of both diseases. Notably, Module 2 was enriched in the Wnt signaling pathway, where nine genes were interconnected (**Figure 4B**). Among them, we found *PIN1* that, not surprisingly, was ranked by Beegle in the top position together with *TP53* (**Supplementary Table S1**). Recent studies have underlined the pivotal role of *PIN1* (peptidylprolyl cis/trans isomerase, NIMA-interacting 1) in the inverse association between cancer and AD (Lee et al., 2011; Driver et al., 2012; Harris et al., 2014; Driver et al., 2015). In Module 2, *PIN1* connected to *GSK3*β and *CTNNB1* genes (**Figure 4B**). *GSK3*β (glycogen synthase kinase 3 beta), a constitutively active protein kinase, acts as a negative regulator in the hormonal control of glucose homeostasis, Wnt signaling, and regulation of transcription factors and microtubules by phosphorylating and inactivating glycogen synthase. In Wnt signaling, GSK3β forms a multimeric complex with APC, AXIN1, and CTNNB1/beta-catenin and phosphorylates the N-terminus of CTNNB1, leading to its degradation mediated by ubiquitin/proteasomes. Neurons derived from iPSCs of sporadic AD reveal elevated Tau hyperphosphorylation, increased amyloid levels, and GSK3β activation (Ochalek et al., 2017). The gene is mostly upregulated in AD studies (Alzbase) and has been associated with AD in GWAS studies (Alzgset). Aberrant nuclear GSK3β may represent a potential target for the clinical treatment of human breast and squamous cell carcinoma (Ugolkov et al., 2018). Altogether, our analysis highlighted autophagy, UPS, and Wnt signaling as molecular mechanisms that could exert an opposite function according to the gene-environment conditions of the specific human tissues.

As a third step of our investigation (**Figure 1**), we considered the key role of miRNAs in the complex regulation of gene expression and their potential pleiotropic action. Focusing on the 37 genes belonging to PPI modules, we identified 12 miRNAs that turned out to be associated with both AD and cancer (**Table 3**). Among these 12 miRNAs, we found several targeting genes involved in autophagy and UPS, including miR-146a-5p (targets *SQSTM1*/p62), miR-34a-5p (targets *BECN1*), and miR-21-5p (targets *STUB1/CHIP*). MiR-146a-5p is upregulated in AD brain and in human neural cells following a number of different stimuli and stresses, including cytokines, β-amyloid, and oxidative stress (Lukiw et al., 2008; Li et al., 2011). In cancer, miR-146a-5p was found acting as both oncogene and oncosuppressor (Li et al., 2010; Pacifico et al., 2010; Hou et al., 2012). Upregulation of miR-146a-5p leads to inflammatory response in AD but has an anti-inflammatory effect in cancer (Lukiw et al., 2008; Cui et al., 2010; Rusca and Monticelli, 2011; Khorrami et al., 2017). Moreover, polymorphisms in the miR-146a gene have been involved in the genetic susceptibility to both AD and cancer (Cui et al., 2014; Xie et al., 2014). MiR-146a-5p targets *SQSTM1*/p62 (Module 1). Of note, p62 can inhibit neuronal death induced by β-amyloid (Geetha et al., 2012) and its inactivation in mice leads to an age-dependent constitutive activation of GSK3β, resulting in hyperphosphorylated tau, neurofibrillary tangles, and neurodegeneration (Ramesh Babu et al., 2008). Taken together, these data suggest that miR-146a-5p upregulation might promote neurodegeneration in AD through inhibition of p62 activity. On the contrary, loss of miR-146a and overexpression of p62 promote cell survival and proliferation in cancer (Fang et al., 2014).

MiR-34a-5p has been widely recognized as a key player in tumor suppression, and its expression is silenced in several cancers (Farooqi et al., 2017; Slabáková et al., 2017). Conversely, miR-34a-5p is overexpressed in AD patients and mouse models; increased miR-34a-5p levels repress genes involved in synaptic plasticity and energy metabolism (Sarkar et al., 2016). MiR-34a knockout also promotes cognitive function in APP/PS1 mice by both inhibiting the amyloidogenic process and increasing synaptic plasticity (Jian et al., 2017; Xu et al., 2018). MiR-34a-5p targets in Module 1 include *BECN1*. The elevated miR-34a-5p levels marking AD condition are expected to reduce the cellular amount of Beclin1 and hence to affect the efficiency of the autophagy machinery in clearing protein aggregates, providing a link between miR-34a dysregulation and β-amyloid pathology in AD. In tumors, the picture is more complicated because autophagy, in general, and Beclin1, in particular, might exert a dual role (White, 2012; Gong et al., 2013). MiR-34a-5p downregulation observed in many tumors would positively affect *BECN1* expression, leading to enhanced autophagy, but the effect of this improved autophagic flux is likely to be dependent on cancer type, cancer stage, and cell type.

MiR-21-5p is frequently overexpressed in various human tumors and cancer stem cells and seems to play an important role in the oncogenic process because it has been associated with high proliferation, invasion, and metastatic potential, as well as with low apoptosis (Pfeffer et al., 2015). Oppositely, miR-21-5p upregulation inhibits cell apoptosis induced by β-amyloid in a GSK3β-dependent way, suggesting a protective role in AD (Feng et al., 2018). Moreover, Tau can enhance miR-21-5p activity (Chauderlier et al., 2018). Because *STUB1* is a miR-21-5p target, it can be speculated that in AD, augmented miR-21-5p levels can exacerbate the neurodegenerative process by reducing STUB1/CHIP activity, leading to less efficient Tau and β-amyloid clearance (Lee et al., 2018). In cancer, miR-21-5p upregulation and consequent *STUB1* downmodulation might have opposite effects, according to the oncogenic or tumor-suppressive effect of *STUB1* (Cao et al., 2016).

**Figure 5:**
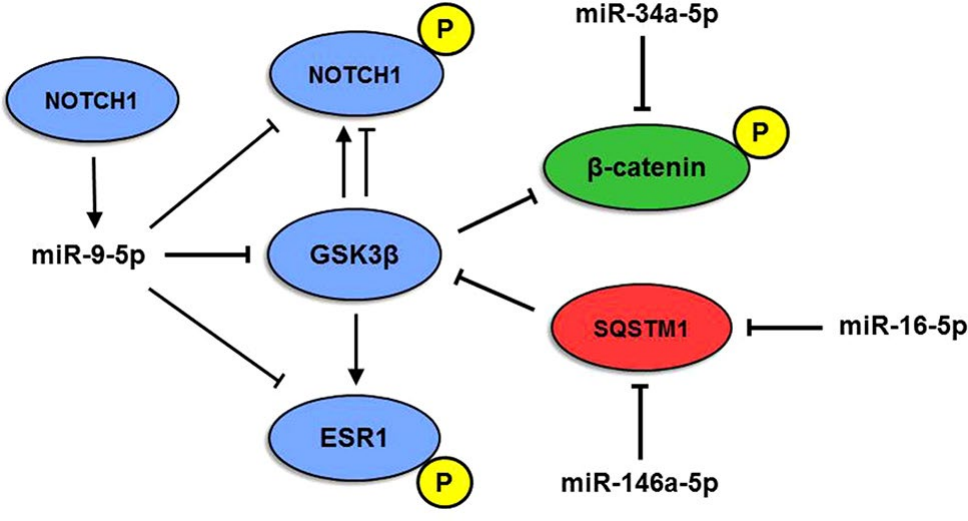
Model of multiple miRNA-gene interactions. The interplay among miR-9-5p, miR-146a-5p, and miR-16-5p and NOTCH1, β-catenin (CTNN1), SQSTM1, ESR1, and GSK3β proteins originates a complex network. **P** indicates the phosphorylated form of the protein. Red, blue, and green colors indicate modules 1, 2, and 3, respectively.

Another interesting miRNA, miR-9-5p, is known as one of the most highly expressed miRNAs in the vertebrate brain and plays a pivotal role in its development (Coolen et al., 2013; Radhakrishnan and Alwin Prem Anand, 2016). MiR-9-5p levels are mostly downregulated in AD (Miya Shaik et al., 2018), whereas both miR-9-5p upregulation and downregulation have been reported in human cancers, where it can either support or suppress tumor development (Nowek et al., 2018). MiR-9-5p targets three genes belonging to Module 2, namely, *NOTCH1*, *GSK3β*, and *ESR1*. Interestingly, upregulation of miR-9-5p and consequent inhibition of the Notch signaling pathway were shown to stimulate neuron differentiation in an APP-overexpressing AD cell model (Li et al., 2017). Here, we propose a complex interplay between miR-9-5p and *NOTCH1*, *GSK3β*, and *ESR1* target genes, also involving genes of Modules 1 and 3 and their miRNAs (**Figure 5**): NOTCH1 is targeted and negatively regulated by miR-9-5p, but its expression, in turn, depends on active Notch signaling (Roese-Koerner et al., 2017). In addition, the GSK3β kinase is able to phosphorylate both NOTCH1 and ER-alpha (encoded by *ESR1*). Phosphorylation by GSK3β can lead to both upregulation and downregulation of NOTCH1 activity (McCubrey et al., 2014), whereas ER-alpha activity is positively regulated by GSK3β (Grisouard and Mayer, 2009). GSK3β also phosphorylates β-catenin, a miR-34a-5p target in Module 3, causing its degradation and leading to suppression of cell proliferation (Mancinelli et al., 2017). On the other hand, GSK3β activity is inhibited by SQSTM1/p62 (Ramesh Babu et al., 2008), a miR-146a-5p and miR-16-5p target belonging to Module 1. Therefore, our analyses highlight the existence of an intricate and highly complex regulatory network between enriched miRNAs and their ALZCAN target genes that may guide future experimental analysis to evaluate their potential role in the inverse occurrence of AD and cancer. Intriguingly, unfolded protein response and metabolism have gained attention as major biological targets for both cancer and AD, thus suggesting the exploitation of cancer drugs repositioning in AD (Monacelli et al., 2016).

Our study has some limitations. The analyses relied on genes spotted by a computer-based approach that considers the scientific literature based on the MEDLINE abstracts and OMIM database, although these databases are highly curated, the list of candidate genes might be incomplete. Second, according to the quality process implemented by Beegle for discovering disease-gene relationship, we assumed all of the 138 genes as true positives, but we cannot exclude the presence of spurious data. Nonetheless, a high percentage of ALZCAN genes (124 out of 138 genes) have been functionally clustered on the basis of significantly enriched GO-biological processes and KEGG terms, thus supporting the text mining analysis in terms of meaningful biological relevance. At the same time, despite various genomics databases included in Metascape, the bioinformatics analysis might be incomplete and other functional PPI modules could be computed. Overall, we are confident that the ALZCAN gene set and the selected miRNAs might be a useful resource for deeper investigation of biological processes underlying the inverse relationship of occurrence between cancer and AD.

## Conclusions

Here, the text-mining tool Beegle was instrumental in extracting a list of 138 candidate genes hidden in the huge amount of biomedical literature. By using the Metascape tool, we obtained significant clusters of molecular and biological processes that might help in the understanding of the intricate set of biological crosstalks between cancer and AD. Through the analysis at the miRNA level, we hypothesized interesting miRNA-gene interrelationships deserving further investigation. By deep investigation of our results, we highlighted three major biological mechanisms: autophagy, UPS, and cell death that might be included in the scenario of the dysregulated processes shared by cancer and AD. Among the ALZCAN gene set and miRNAs, we proposed nine genes (*SQSTM1*, *UCHL1*, *STUB1*, *BECN1*, *CDKN2A*, *TP53*, *EGFR*, *GSK3Β*, and *HSPA9*) and five miRNAs (miR-146a-5p, MiR-34a-5p, miR-21-5p, miR-9-5p, and miR-16-5p) as best candidates that warrant further investigation. Recently, the new avenue of drug repositioning has been proposed for the treatment, so far poorly effective, of AD. In this light, we believe that the comprehensive exploitation of the data here presented might provide potential insights for identifying innovative therapeutic approaches for AD.

## Data Availability

All datasets analyzed for this study are included in the manuscript and the **Supplementary Files**.

## Author Contributions

Full access to all of the data in the study and taking responsibility for the integrity of the data and the accuracy of the data analysis: CB. Study concept and design: CB. Acquisition of data: CB, MV, FA, NJ, and AS. Analysis and interpretation of data: CB and MV. Drafting of the manuscript: CB. Critical revision of the manuscript for important intellectual content: CB, FA, MM, and GB. Text mining and statistical analysis: CB, MV, AO, RS, and AS. Study supervision: CB. All authors read and approved the final manuscript.

## Conflict of Interest Statement

The authors declare that the research was conducted in the absence of any commercial or financial relationships that could be construed as a potential conflict of interest.

## Supplementary Material

Figure S1 Heat-map of the GTEx data for 138 ALZCAN gene set. The rows correspond to 53 human tissues and the columns to 138 genes. See **Supplementary Table S2** for more details.

Table S1 Description of ALZCAN gene set. The table includes the following columns: Ensembl ID, Entrez Gene ID, Gene Symbol, description, BEEGLE Gene Rank, ALZGSET flag, AlzDEGs (total studies), AD DEGs (Up), AD DEGs (Dn), CGC flag, HPA prognostic flag, TWAS flag, longevity flag.

Table S2 The gene expression levels of 138 ALZCAN genes across 53 human tissues (GTEx database). Gene expression data are reported as raw data of Reads Per Kilobase per Million mapped reads (RPKM).

Table S3 The HPA annotation of 138 ALZCAN genes for both normal and cancer human. The table includes the following columns: gene name, ensembl ID, protein class, prognostic p-value, prognostic gene flag, cancer tissues (protein level detection), normal tissues (manual protein level detection), RNA cancer category (HPA classification) RNA tissue category (HPA classification).

Table S4 List of GO and KEGG terms obtained by functional enrichment terms (Metascape).

Table S5 GO-MCODE modules (Metascape).

Table S6 List of miRNAs targeting ALZCAN gene set and annotation (ToppGene).
