# Supplementary figures and images for: Candidate Genes and MiRNAs Linked to the Inverse Relationship Between Cancer and Alzheimer’s Disease: Insights From Data Mining and Enrichment Analysis
Source: Front Genet. 2019 Sep 24;10:846. doi: 10.3389/fgene.2019.00846 (PMC6771301; doi:10.3389/fgene.2019.00846)

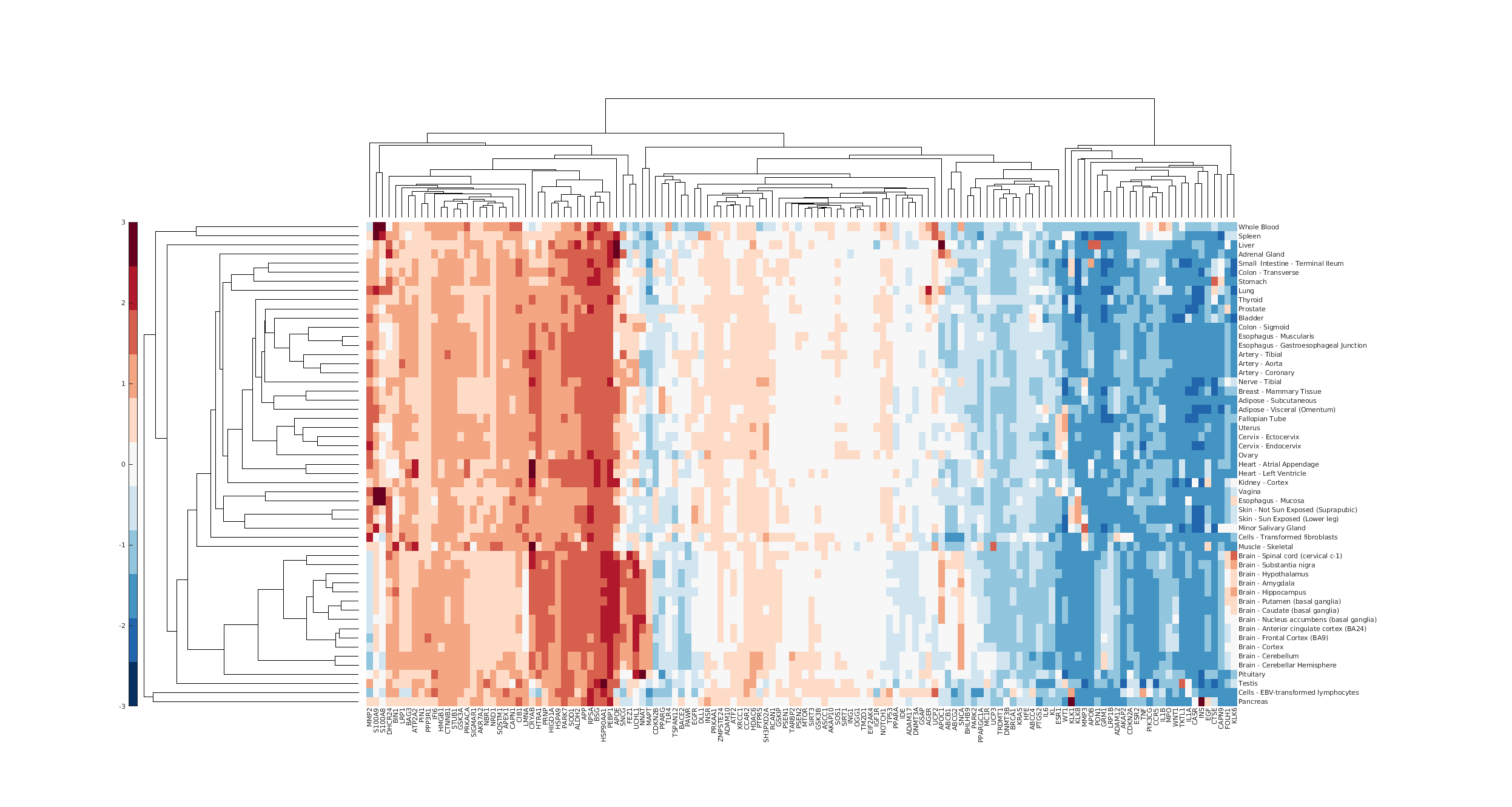

Supplement: Figure S1 — Heat-map of the GTEx data for 138 ALZCAN gene set. The rows correspond to 53 human tissues and the columns to 138 genes. See Supplementary Table S2 for more details. [file Image_1.png]
